# Ground Reaction Forces and Center of Pressure within the Paws When Stepping over Obstacles in Dogs

**DOI:** 10.3390/ani12131702

**Published:** 2022-06-30

**Authors:** Danae Charalambous, Therese Strasser, Alexander Tichy, Barbara Bockstahler

**Affiliations:** 1 Department of Companion Animals and Horses, University Clinic for Small Animals, Small Animal Surgery, Section for Physical Therapy, University of Veterinary Medicine, 1210 Vienna, Austria; therese@strasser-org.at (T.S.); barbara.bockstahler@vetmeduni.ac.at (B.B.); 2 Department of Biomedical Sciences, Platform for Bioinformatics and Biostatistics, University of Veterinary Medicine, 1210 Vienna, Austria; alexander.tichy@vetmeduni.ac.at

**Keywords:** canine physical therapy, dog, gait analysis, center of pressure, obstacle crossing, cavaletti, leading limb, trailing limb

## Abstract

**Simple Summary:**

Physical therapy and rehabilitation are emerging in veterinary medicine, and more research is needed to understand the effect of various exercises on kinematics and kinetics in animals. This will allow the animal physiotherapist to best utilize these exercises as a therapeutic and even diagnostic tool. Walking over obstacles is a typical canine physiotherapy exercise; however, no studies investigating the kinetics have been conducted. The present study showed significant changes in ground reaction forces and center of pressure in dogs walking over obstacles compared to normal walking. This can reflect a challenge that the animals have to overcome in order to perform this exercise. The data can be used for further studies in diseased animals or in the future as a diagnostic tool.

**Abstract:**

Walking over obstacles is a widely used physiotherapy exercise in dogs. Current research is limited to the effect of this exercise in kinematics and muscle activation in dogs. The present study assessed the influence of walking over obstacles on the ground reaction forces (GRFs) and center of pressure (COP) in dogs. Data of dogs walking over one and two obstacles over a pressure platform were retrospectively analyzed and compared to normal walking. Walking over one obstacle did not affect the GRFs and COP of the forelimbs; however, significant changes were observed for the hindlimbs, especially the leading hindlimb. Walking over two obstacles caused significant changes to only one value at the forelimbs, whereas multiple significant changes in the GRFs and COP values were observed at the hindlimbs. Walking over obstacles seems to be challenging even for healthy adult dogs. Further studies are needed to investigate how different heights of obstacles and distances between them can further challenge the animals. The combination of kinetics and kinematics during walking over obstacles may be used in future as a diagnostic tool in geriatric and neurological patients in order to assess their proprioception awareness or to assess the improvement after an intervention, e.g., physiotherapy treatment.

## 1. Introduction

Physical therapy and rehabilitation are emerging in veterinary medicine and are currently recognized as a vital part of treatment strategy in many morbidities. Since the 1980s, canine physical therapy started to gain clinical attention, and it has demonstrated tremendous scientific advances. One study reported that approximately 70% of veterinarians in United States refer animals for physiotherapy [1].

Physiotherapy’s main purpose is to maintain, promote, and restore optimal function, optimal fitness, wellness, and quality of life [2]. The veterinary physiotherapist can utilize a plethora of available modalities and therapeutic exercises to achieve the best functional outcome for animals [2,3,4].

One of the most common prescribed exercise in veterinary physiotherapy is walking over obstacles or cavaletti poles (rails). A series of rails are placed perpendicular to the animal’s direction of movement and the animal is guided over them. The distance between the poles and their height depends on the size and skill level of the animal [3]. Other parameters can also be altered so the animal can be challenged further. For example, adjustments of the direction of the poles (e.g., in a circle), variable heights and spacing, and addition of a wobble cushion. The aim of this exercise is to increase the range of motion (ROM) of certain joints, endorse limb loading, lengthen the strides in all limbs, activate atrophic muscles, and promote proprioception, balance, and coordination [2,3]. Walking over obstacles can be used in a variety of diseased and healthy animals, including for osteoarthritis, neurological and orthopedic conditions (surgical and nonsurgical), or fitness and strengthening purposes, in both canine pets and athletes [3].

Walking over obstacles also resembles an everyday motor task for both animals and humans. Walking over various obstacles, e.g., stones and branches, is essential in order to interact adaptively with the environment [5]. For healthy individuals, it rarely poses a challenge; however, for elderly people [6] and people with neurologic conditions, it can be challenging. The same principle may apply for animals. Obstacle crossing has been studied in humans with developmental coordination disorder and Parkinson’s disease, as well as, children with cerebral palsy stroke, in order to investigate the dynamic balance strategies in these groups and, in some cases, to differentiate people at risk of falls [7]. In humans, walking over obstacles is also a functional test to assess impairment with traumatic brain [8] and spinal cord injury [9].

Kinematic and kinetic measurements have been used in order to investigate the influence of obstacle crossing in humans [5,6,10]. In dogs, researchers investigated the impact of cavaletti rails on the ROM of healthy [11] and diseased [3,12] dogs and on the activation of specific muscles through electromyography (EMG) [13,14] but not on the kinetics. In veterinary medicine, interest in the measure of ground reaction forces (GRFs) with force or pressure plates has been increasing gradually over the past few decades. It provides objective data on the forces created between the limb and the ground during the stance phase, in a noninvasive manner. It has been used as a method of normal movement assessment in various healthy animals [15,16,17,18], as an objective diagnostic tool for lameness [19,20], and to evaluate various treatments [21,22,23,24]. Many parameters can be obtained during a gait analysis, including peak vertical force (PFz), which is the maximum force exerted in the vertical direction, vertical impulse (IFz), which is the area under the force–time curve that takes into account the force and contact time [25], and the time to PFz, which is the time during the stance phase where PFz is reached (TPFz), for each limb [25,26].

Center of pressure (COP) is another variable that can be investigated during gait analysis. COP is the location where the instantaneous vector of the ground reaction forces acts. During ground contact, the position of the COP changes continuously, thus creating the COP path [27]. Analysis of the COP provides a dynamic reflection of global locomotion and postural control [28], and it has been used to quantify various gait abnormalities [29]. In dogs, COP has been investigated in healthy animals [30], in animals with orthopedic [31,32] and neurological conditions [29], and to test the efficacy of a treatment [33]. The COP may be a useful tool to obtain information regarding biomechanical modifications and postural control during exercises in healthy and impaired animal.

While, as described, research has shown the influence of obstacle crossing on the joint kinematics, no information is available on the effect on ground reaction forces and postural stability of dogs.

The aim of this study was, therefore, to investigate if walking over obstacles affects the postural control of the dogs and the ground reaction forces of each limb by measuring the GRFs and COP of healthy dogs walking over one and two obstacles at the same height. Our hypothesis was that ground reaction forces and COP values would be influenced by crossing obstacles, indicating higher forces acting on the limbs and higher COP values.

## 2. Materials and Methods

### 2.1. Ethics

Data were retrospectively evaluated from previous gait analysis measurements of 10 Labrador Retrievers at the Veterinary University of Vienna [34,35]. During the measurements for the previously mentioned study, the animals walked over one and two obstacles, which was approved by the Institutional Ethics Committee in accordance with guidelines for good scientific practice and with national legislation (10/09/97/2011).

### 2.2. Animals and Exclusion Criteria

To be included in the mentioned study, the animals had to present a normal orthopedic and neurological examination. Furthermore, a symmetry index (SI) less than 3% was required, as a symmetry index of up to 3% is considered normal for dogs and values higher than 3% are considered to indicate lameness [27,36,37]. At least four valid passes for each limb (during obstacle measurements) were required for a dog to be included in the study, where valid passes over the obstacles were those where the animals did not touch or drop the obstacles, turn their head, or pull on the lead. With respect to the inclusion criteria, eight dogs (five females and three males) were evaluated for the presented study. The mean age was 40.75 ± 26.28 months old (median = 32 months, minimum = 13 months, maximum = 89 months), and the mean weight was 27.94 ± 3.48 kg (median = 27.25 kg, minimum = 24 kg, maximum = 36 kg).

### 2.3. Equipment

A pressure measurement plate (FDM Type 2 from Zebris Medical GmbH, Allgäu, Germany) with dimensions of 203 × 54.2 cm, 15,360 piezoelectric sensors, and a sampling rate of 100 Hz was used. The plate was covered with a black, 1 mm thick rubber mat made out of polyvinylchlorid to avoid slipping. In order to be able to assign the measured values to the correct limb of the respective test during data evaluation, each measurement run was filmed with a Panasonic camera, model NV-MX500. The data were gathered using WinFDM software (v1.2.2, Zebris Medical).

### 2.4. Measurement Procedure

The dogs were first allowed to get used to the examination room before the measurement. For this purpose, they were allowed to move freely in the room. As soon as the dogs became accustomed to the environment, they were subjected to a standard analysis of the ground reaction forces, as described by Reicher et al. [27], before walking over obstacles. The dogs were led on the left side of the handler, as in everyday life, and always from the same direction at their comfortable speed for walking. This was repeated until a sufficient number of valid steps were collected. Valid passes were those where the animals did not turn their head, pull on the lead, or change speed. At least four valid passes were analyzed for normal walking. After normal walking, the animals were guided to walk over one and two obstacles by their owner; one or two cylindrical yellow polyvinyl chloride (PVC) poles with diameter of 2.5 cm and length of 1 m were placed on a cone to achieve a height of 13 cm (for both conditions). The distance between the obstacles was 35 cm for the condition of walking over two obstacles, which allowed all animals to take one step in between the obstacles. The obstacles were long enough such that the cones did not come into contact with the measurement plate.

The animals were walked over each obstacle conditions six times. If, during the first trial, at least four valid steps were not obtained (see Section 2.2), the measurement was repeated 1 week later.

### 2.5. Data Analysis

#### 2.5.1. Software

The data were analyzed with the custom software Pressure Analyzer (Michael Schwanda, version 4.6.5.0) and then exported to Microsoft^®^ Excel^®^ 2016. The individual footprints recorded during the valid passes were manually assigned to the corresponding limb with the help of the recorded video.

#### 2.5.2. Assignment of the Limbs

##### Normal Walking

The standard method of assessment included the identification and assignment of each limb as the right forelimb (RFL), left forelimb (LFL), right hindlimb (RHL), and left hindlimb (LHL).

##### Walking over One Obstacle

**Figure 1:**
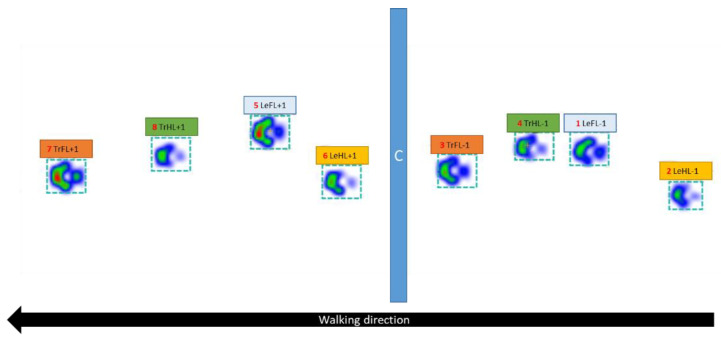
Labeling of the limbs when crossing a single cavaletti (dog 1). Blue—leading forelimb, yellow—leading hindlimb, orange—trailing forelimb, green—trailing hindlimb; −2—first stance phase before the cavaletti, −1—second stance phase before the cavaletti, +1—first stance phase after the cavaletti. The red numbers indicate the order in which the paws touched the plate.

Each stance phase of all limbs before and after the obstacle was assessed individually (Figure 1). The leading forelimb (LeFL) was the forelimb that touched the ground first after the obstacle, and the trailing forelimb (TrFL) was the forelimb that touched the ground second after the obstacle. The same principle was applied for the hindlimbs, whereby the hindlimb that touched the ground first after the obstacle was the leading hindlimb (LeHL), and the hindlimb that touched the ground second was the trailing hindlimb (TrHL). Furthermore, each leading and trailing limb was assigned a number. The number next to each limb represents the position of the limb relative to the obstacle, with minus values indicating limbs before the obstacle and positive values indicating limbs after the obstacle (Figure 1). For example, −1 denotes the limb 1 stance phase before the obstacle, whereas +1 denotes the limb 1 stance phase after the obstacle. Some limbs were not assigned a value of −2 or +2 because the limb was not placed on the pressure plate. For comparison with normal walking, the leading forelimb was compared to the right forelimb, the trailing forelimb was compared to the left forelimb, the leading hindlimb was compared to the right hindlimb, and the trailing hindlimb was compared to the left hindlimb (Table 1).

##### Walking over Two Obstacles

**Figure 2:**
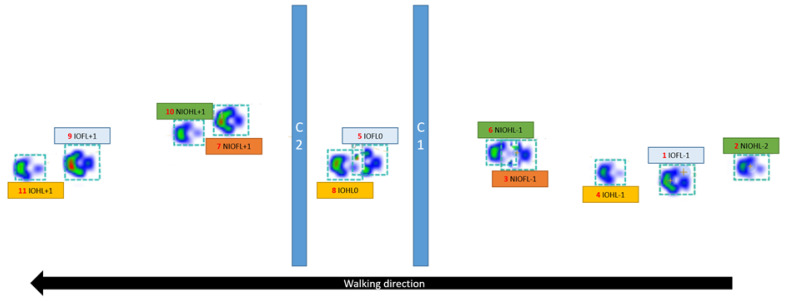
Labeling of the limbs when crossing two cavaletti (dog 1). Blue—forelimb which stepped between the obstacles, yellow—hindlimb which stepped between the obstacles, orange—forelimb which did not step between the obstacles, green—hindlimb which did not step between the obstacles; 0—stance phase between the obstacle, −2—first stance phase before the cavaletti, −1—second stance phase before the cavaletti, +1—first stance phase after the cavaletti. The red numbers indicate the order in which the paws touched the plate.

For this condition, inter-obstacle (IO) limbs were those which stepped between the two obstacles during walking (IOFL for the forelimbs and IOHL for the hindlimbs), whereas non-inter-obstacle (NIO) limbs were those which did not pass between the obstacles (NIOFL for the forelimbs and NIOHL for the hindlimbs). Similarly to the condition of walking over one obstacle, numbers were assigned to each limb according to their position relative to the obstacles (Figure 2). The reference point was the limb between the obstacles, which was assigned the number zero. The stance phases of the limbs before the first obstacle were assigned negative numbers, while the stance phases of the limbs after the second obstacle were assigned positive numbers (Figure 2). Some limbs were not assigned a value of −2 or +2 because the limb was not placed on the pressure plate. For comparison with normal walking, the IO forelimb was compared to the right forelimb, the NIO forelimb was compared to the left forelimb, the IO hindlimb was compared to the right hindlimb, and the NIO hindlimb was compared to the left hindlimb (Table 2).

### 2.6. Parameters under Investigation

The following parameters were used for evaluation:

- Mean speed (m/s) and acceleration (m/s^2^), calculated on basis of the left forelimb by the software of the pressure plate;
- Peak vertical force (PFz in N);
- Vertical impulse (IFz in N/s), describing the impulse in the *Z*-direction;
  ○ The PFz and the IFz were normalized using the following formula and expressed as %TF:
    $$\mathrm{Value}\;\mathrm{in}\;\%\;\mathrm{of}\;\mathrm{total}\;\mathrm{force}=\frac{\mathrm{XFzFL}\;}{\left(\mathrm{XFzFL}+\mathrm{XFzFR}+\mathrm{XFzHL}+\mathrm{XFzHR}\right)}\times 100,$$
    where XFz is the mean value of PFz or IFz of the valid steps, FL is the left forelimb, FR is the right forelimb, HL is the left hindlimb, and HR is the right hindlimb.
- Mean duration of the stance phase (StPh) in seconds;
- Time of occurrence of PFz (TPFz) as a percentage of the stance phase of the respective limb;
- Asymmetry index (ASI):
  $$\mathrm{SIXFz}\;\left(\%\right)=\mathrm{abs}\left(\frac{\left(\mathrm{XFzLF}-\mathrm{XFzRF}\right)}{\left(\mathrm{XFzLF}+\mathrm{XFzRF}\right)}\right)\times 100,$$
  where XFz is the mean value of PFz or IFz of valid steps, HL is the left hindlimb, and HR is the right hindlimb; perfect symmetry between the right and left hindlimbs was assigned a value of 0%.

The evaluation of the COP was performed according to Reicher et al. [27] as follows:

- Mediolateral and craniocaudal COP displacement: These parameters are the differences between the maximum positive and negative COP values along the mediolateral and craniocaudal axes, respectively. The mediolateral displacement was normalized to the maximum width of the paw contact area (COPmed-lat%), while the craniocaudal displacement was normalized to the maximum length of the paw contact area (COPcran-caud%).
- COP-Area: The COP area is a measurement of the area covered by the COP movement. It was normalized to the paw contact area and expressed as a percentage (COP-Area%).
- COP-Speed: The COP speed is the mean speed of the movement of the COP (COP-Sp, mm/s).
- COP-Radius: The COP radius is the mean distance of all COP points to the center point of all COP points. This parameter was also normalized to the paw contact area and given as a percentage (COP-Radius%).
- Speed and Acceleration: The acceleration and speed when walking over obstacles was calculated on the basis of the left forelimb by the software of the pressure plate.

### 2.7. Statistical Analysis

All data were analyzed using IBM SPSS v27. In each condition (walking over one obstacle, walking over two obstacles), the parameters (acceleration, speed, PFz, IFz, StPh, TPFz, COPmed-lat%, COPcran-caud%, COP-Area%, COP-Sp, and COP-Radius%) measured for each limb (leading/trailing, inter-obstacle/non-inter-obstacle) in each step position were compared to those measured the corresponding limb in the normal walking condition using linear mixed-effects models. Multiple comparisons were performed using Sidak’s alpha correction procedure. A *p*-value below 5% (*p* < 0.05) was considered significant.

## 3. Results

### 3.1. Walking over One Obstacle

#### 3.1.1. Forelimbs

**Figure 3:**
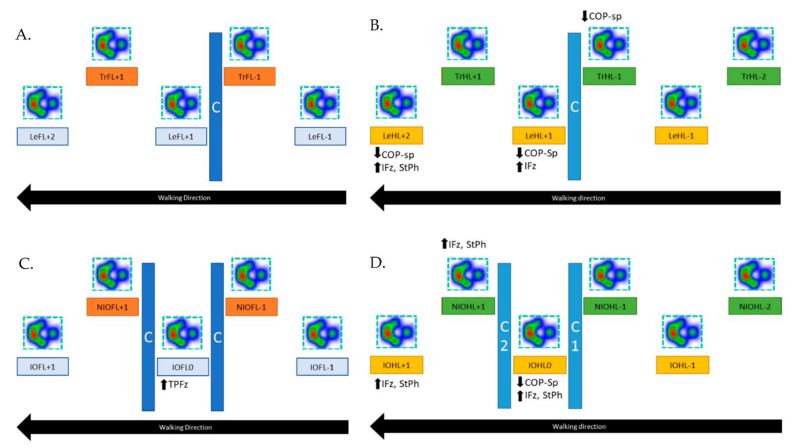
Significant differences between walking over one or two obstacles compared with normal walking are shown. (**A**) Results of the forelimbs during walking over one obstacle. (**B**) Results of the hindlimbs during walking over one obstacle. (**C**) Results of the forelimbs during walking over two obstacles. (**D**) Results of the hindlimbs during walking over two obstacles. COP-sp, COP speed; IFz, vertical impulse; StPh, stance phase; TPFz, time to peak vertical force. All mean values, standard deviation and *p*-values are given in Appendix A.

No significant differences were detected regarding the ground reaction forces and COP parameters (Figure 3A).

#### 3.1.2. Hindlimbs

The leading hindlimb showed, in both stance phases after the cavaletti (LeHL+1 and LeHL+2), a significantly higher IFz (%TF) than the reference limb during normal walking (*p* = 0.012 and *p* = 0.041, respectively). This was accompanied by a significantly longer StPh of the second stance phase after the obstacle (*p* = 0.015). In both stance phases, a significantly lower COP-Speed was found (*p* = 0.012, *p* = 0.007, respectively). Furthermore, the COP-Speed of the trailing hindlimb in the stance phase directly before the cavaletti (TrHL−1) was significantly lower (*p* = 0.023) compared to the reference hindlimb during normal walking (Figure 3B).

### 3.2. Walking over Two Obstacles

#### 3.2.1. Forelimbs

Compared to the reference limb during normal walk, the forelimb that stepped in between the obstacles (IOFL0) showed a significantly later occurrence of PFz (*p* = 0.018) (Figure 3C).

#### 3.2.2. Hindlimbs

Compared to the reference limb during normal walking, the hindlimb that stepped in between the two obstacles showed a significantly higher IFz (%TF) during the stance phase in between (IOHL0, *p* = 0.007) and directly after (IOHL+1, *p* = 0.012) the obstacles. Both stance phases were accordingly significantly longer (IOHL0 *p* = 0.030, IOHL+1 *p* = 0.018). During the first stance phase after the obstacle, the limbs also displayed a significantly lower COP-Speed (*p* = 0.005). The hindlimb that did not step between the cavaletti displayed a significantly higher IFz (%TF) at the first stance phase after the obstacles (*p* = 0.023), as well as a longer stance phase (*p* = 0.047) (Figure 3D).

### 3.3. Speed and Acceleration

Walking over two obstacles resulted in a significantly lower speed (*p* = 0.021) compared to normal walking.

## 4. Discussion

Our hypothesis was that ground reaction forces would reflect a higher load on the limbs and that COP values would show an increase while crossing obstacles. These hypotheses were only partially confirmed for IFz (%TF) for the leading hindlimb in the one-obstacle condition and the hindlimbs in the two-obstacle condition. This increase in IFz values was probably caused by an increase in the stance phase duration. The only influenced COP value was its speed, displaying lower values compared to normal walking in the trailing hindlimb before crossing one obstacle and directly after the obstacles in the limb that stepped in between the two obstacles.

Major findings of our study were that the forelimbs did not show the same changes in GRFs and COP values when animals were crossing either one or two obstacles compared to the hindlimbs. When crossing one obstacle, the leading limb showed more changes compared to the trailing limb. These changes were observed for the first and second stance phases after the obstacle. Lastly, when crossing two obstacles, where leading limbs (the limb that cross the obstacle first) alternated, changes were observed in both hindlimbs between and after the obstacles.

In bipedal locomotion, there is only one leading and one trailing limb; thus, direct correlations between human and animal locomotion studies are naturally problematic. However, research has shown that differences in kinematics, kinetics, and EMG exist between leading and trailing limbs in humans and rats during obstacle clearance [5,6,38,39,40,41,42,43,44]. In a study where kinematics and EMG were performed in rats, the toe trajectory of the leading forelimb was observed to be more accurately regulated than that of the trailing forelimb [38].

Two of the hypotheses that have been proposed to explain this difference in trajectory during kinematic assessment of the leading and training limbs in humans are vision pre-programming [41] and information transfer between legs [40]. During locomotion, the leading limb is visible in the peripheral visual field; however, the individual must rely on memory of the obstacle to guide the trailing limb [44]. One report indicated that individuals sometimes have difficulty in controlling the movement of the trailing limb due to loss of visual feedback [45]. In quadrupeds, it has been proposed that parietal cortical areas are associated with movement planning and working memory to guide the hindlimbs over an obstacle previously cleared by the forelimbs [46]. Studies in horses [47] and cats [46,48] have been conducted regarding the memory of obstacles. In cats, it was shown that there is a long-lasting memory of obstacles (up to 10 min), which includes information of the size and position of the obstacle relative to the animal [48]. Horses not only displayed memory of an obstacle as measured by the hindlimb being lifted over the obstacle for durations up to 15 min, but it was also shown that previous experience of stepping over an obstacle led to pause-related hindlimb lifting at the location where the object was previously located, even in trials for which there was no obstacle and no previous forelimb lifting. The authors concluded that hindlimb obstacle clearance is guided by place–object memory, which can guide hindlimb stepping, as well as overshadow working memory from forelimb stepping [47].

In a human study, people walked over an obstacle 25 times, before the obstacle was removed; then, subjects were instructed to step over the obstacle as if it was still there. Kinematic assessment showed that action was impaired for both limbs when guided by obstacle height memory (crossing the obstacle as if it was there), but action was impaired to a greater extent for the trailing limb. The authors concluded that viewing the obstacle during approach seems to facilitate the memory needed to guide obstacle crossing, particularly for the trailing limb [44].

This information can maybe explain some of the results of this study. If dogs rely on working or place–object memory for the clearance of obstacles with the hindlimbs, they may need more time to accurately execute the action. This can explain why we found more differences in the hindlimbs compared to the forelimbs, as well as increases in IFz and StPh. The IFz is the area under the force–time curve taking both force and contact time of the limb into consideration [25], whereas StPh is the duration of the stance phase; these are time-dependent values. Even though animals have leading and trailing limbs for both forelimbs and hindlimbs, the animal only has visual contact with the leading forelimb. Perhaps all other limbs are comparable with the trailing limb of humans. Moreover, in our study, we observed that LeHL+2 when crossing one obstacle had similar adaptations to LeHL+1, while IOHL+1 had similar adaptations to IOHL0. On the basis of this knowledge, it can be speculated that animals continue to use the same adaptations even after crossing the obstacles. However, more stance phases after the obstacles are needed to confirm this hypothesis. Further studies in animals should investigate if walking over obstacles has an implication in improving hindlimb proprioception and awareness.

As mentioned above, in human and rat studies, differences between leading and trailing limbs were revealed. In our study, we found more changes in the leading limb compared to the trailing limb when crossing one obstacle. Another kinematic study in rats found that, when stepping over the obstacle, the toe trajectories of the forelimbs and hindlimbs when functioning as the leading limb differed from those when functioning as the trailing limb [43]. The same results were found in adult and elderly people (the trajectory of the leading leg was different from that of the trailing leg when passing over obstacles) [42].

Differences in kinetic strategies during obstacle crossing were also observed regarding the leading and trailing limbs in humans [5,6,39]. In our study, we found a significant increase in the IFz of the leading hindlimb (LeHL+1 with one obstacle), IOHL0, and NIOHL+1. IOHL0 is the leading hindlimb of the first obstacle when walking over two obstacles. However, for the second obstacle, NIOHL+1 is the leading hindlimb. In a human study, among other results, it was found that the leading limb had higher impulse when crossing an obstacle compared to no obstacle. However, this was also applicable for the trailing limb [6]. Another study by Wang et al. in humans, investigating the effect of the distance between two obstacles by using force plate, revealed that the foot integrated pressure (equivalent to IFz) was significantly higher in the leading limb than the trailing limb when the distance between two obstacles was one step [10]. Further research is warranted to investigate if walking over obstacles can influence the IFz in dogs with orthopedic diseases, where this value is reduced [24].

Moreover, higher StPh was observed at the IOHL0 (leading limb for the first obstacle) and NIOHL+1 (leading limb for the second obstacle) when walking over two obstacles. The StPh of LeHL+1 when crossing one obstacle was higher than when walking, without reaching significance (*p* = 0.08). The changes observed for the leading limb regarding forces and force–time characteristics were a result of not only landing after an obstacle but also controlling the trailing limb over obstacles [6]. Furthermore, when walking over two obstacles, the trailing limb must overcome two obstacles; thus, the swing phase of this limb may be longer than that of the leading limb that must overcome just one obstacle [10]. In a human study, it was observed that subjects spent a longer time on the leading limb than the trailing limb during obstacle crossing [45]. Sparrow et al. [49] and Chen et al. [39] also found that the crossing speed decreased and step duration increased across different obstacle heights, and both these variables increased significantly compared to unobstructed walking. If the same principles are applied to diseased animals, this exercise can be useful in dogs where the aim is to increase the StPh.

Another finding of our study is that that the PFz was reached later in the stance phase at IOFL0. In the previously discussed study [6], the researchers found by using a force plate that walking over a high obstacle required a longer time to peak force, increased force, and increased impulse in the leading and trailing limbs [6].

In our study, we found that the speed when walking over two obstacles was significantly lower than during normal walking. In a study investigating the kinetic characteristics, by using a force plate, of the leading and trailing limbs during normal walking and stepping over obstacles of three different heights (10%, 20%, and 30% of leg length), a significant decrease in crossing speed between conditions was observed [5]. Furthermore, as mentioned above, Sparrow et al. [49] and Chen et al. [39] found that crossing speed decreased and step duration increased across different obstacle heights, and both these variables increased significantly compared to unobstructed walking. This is perhaps because the animals needed more time to navigate the obstacles compared to normal walking.

The only significant difference that we found regarding COP values was the speed of the COP, which represents the mean speed between all COP points of each paw. These significant differences were observed only at the hindlimbs. An increase in COP values can be interpretated as a sign of reduced stability [31,32] and biomechanical adaptations [29]. A study by Wang and Watanabe [50] investigated the COP in humans during obstacle crossing of various heights. The comparison of our results with this study is again difficult because, in that study, the COP was divided into four phases (loading response phase, mid-stance phase, terminal stance phase, and pre-swing phase), and the results were different for each phase. However, they found lower COP velocities with higher obstacles; the authors suggested that “this reduction in COP velocity may indicate a control strategy to keep a smooth progression of the body over the stance foot when stepping over higher obstacles”. Even though only one obstacle height was investigated in our study, it is possible that this decrease in COP speed was a strategy for a smooth clearance of the obstacles. Lower COP speed may represent a more controlled movement of the limb. This exercise may be useful in dogs where the aim is to train proprioception and balance. The absence of significant differences of the other COP parameters could be an indication that walking over obstacles does not change the limb support in healthy dogs. Lopez et al. [31] described that higher values of the caudal margin, limb COP pathway length, craniocaudal index (similar to COPcran-caud%) and center of the pressure excursion index (similar to COPmed-lat%) are associated with better limb support. However, further studies are warranted to investigate if walking over obstacles can influence the caudal margin, as we did not measure this value and the influence of obstacles in diseased dogs on the COP values.

Some of the limitations of this study were its retrospective nature and small sample size. Moreover, we included only Labrador Retrievers in this study. Dogs can be characterized as ectomorphic, endomorphic, and mesomorphic according to their body conformation [4], and it has been shown that differences exist between breeds regarding kinetics and kinematics [17,51]. In a study comparing Labrador Retrievers and German Shepherds, among other differences, they found that the body center of pressure of Labrador Retrievers was located more cranially than in German Shepherds [17]. Additionally, it has been proven that, even within the same breed, differences exist in kinematics and kinetics [52,53]. Another limitation of this study is that we recorded different velocities during normal walking and walking over two obstacles. We could not adjust the speed in the two conditions because the animals needed more time to cross the obstacles; on the other hand, we could not force the animals to walk more slowly during normal walking, because this would not represent a comfortable speed for walking. Current guidelines for minimizing variability are to use a narrow velocity range (±0.3 m/s) with controlled acceleration (±0.5 m/s^2^) [54]. In our study the speed, was 1.06 ± 0.12 m/s during normal walking and 0.90 ± 0.1 m/s during walking over two obstacles.

It has been also proven that differences in velocity of 1.5–2.2 m/s during trotting in dogs do not alter the GRFs [54]. We also investigated only one height of obstacle and only one distance between obstacles. Further studies are needed to understand how heights and distances affect the animals. Lastly, we examined only the vertical forces and not the mediolateral and craniocaudal forces, which could yield more information. The combination of kinematics and kinetics could also provide a more in-depth understanding of the changes during walking over obstacles.

Furthermore, the long-term outcome of investigating kinematics and kinetics when stepping over obstacles will provide useful information for training healthy and impaired animals and can be utilized in order to assess impaired gait patterns associated with aging, injury, or other disabling conditions [5], as well as an evaluation criterion of motor function in dogs recovering from an injury.

## 5. Conclusions

Our results showed significant differences when the dogs were walking over obstacles compared to normal walking, especially at the hindlimbs. Walking over obstacles seems to be challenging even for healthy adult dogs. The dogs increased the impulse and stance phase, while they reduced the COP-Sp in order to overcome the obstacles without touching them or falling. Further studies are needed to understand the fundamental adaptive mechanism (or avoidance strategies) of applying forces to move the body effectively and safely over obstacles and to investigate how different heights of the obstacles and distances between them can further challenge the animals. Moreover, the combination of kinetics and kinematics during walking over obstacles may be used in future as a diagnostic tool in geriatric and neurological patients in order to assess their proprioception and awareness or to assess improvement after an intervention, e.g., physiotherapy treatment.

## Figures and Tables

**Table 1:** Description of the abbreviations used during walking over one obstacle and the limbs with which they were compared during normal walking.

Limb during Walking over One Obstacle	Position Relative to the Obstacle	Description of the Position Relative to the Obstacles	Abbreviation	Limb during Normal Walking for Comparison
Leading forelimb	−1	The stance phase before the obstacle	LeFL−1	Right forelimb
Leading forelimb	+1	The stance phase after the obstacle	LeFL+1	Right forelimb
Leading forelimb	+2	The second stance phase after the obstacle	LeFL+2	Right forelimb
Trailing forelimb	−1	The stance phase before the obstacle	TrFL−1	Left forelimb
Trailing forelimb	+1	The stance phase after the obstacle	TrFL+1	Left forelimb
Leading hindlimb	−1	The stance phase before the obstacle	LeHL−1	Right hindlimb
Leading hindlimb	+1	The stance phase after the obstacle	LeHL+1	Right hindlimb
Leading hindlimb	+2	The second stance phase after the obstacle	LeHL+2	Right hindlimb
Trailing hindlimb	−2	Two stance phases before the obstacle	TrHL−2	Left hindlimb
Trailing hindlimb	−1	The stance phase before the obstacle	TrHL−1	Left hindlimb
Trailing hindlimb	+1	The stance phase after the obstacle	TrHL+1	Left hindlimb

**Table 2:** Description of the abbreviations used during walking over two obstacles and the limbs with which they were compared during normal walking.

Limb during Walking over Two Obstacles	Position Relative to the Obstacles	Description of the Position Relative to the Obstacles	Abbreviation	Limb during Normal Walking for Comparison
Inter-obstacle forelimb	−1	The stance phase before the first obstacle	IOFL−1	Right forelimb
Inter-obstacle forelimb	0	The stance phase in-between the two obstacles	IOFL0	Right forelimb
Inter-obstacle forelimb	+1	The stance phase after the second obstacle	IOFL+1	Right forelimb
Non-inter-obstacle forelimb	−1	The stance phase before the first obstacle	NIOFL−1	Left forelimb
Non-inter-obstacle forelimb	+1	The stance phase after the second obstacle	NIOFL+1	Left forelimb
Inter-obstacle hindlimb	−1	The stance phase before the first obstacle	IOHL−1	Right hindlimb
Inter-obstacle hindlimb	0	The stance phase in-between the two obstacles	IOHL0	Right hindlimb
Inter-obstacle hindlimb	+1	The stance phase after the second obstacle	IOHL+1	Right hindlimb
Non-inter-obstacle hindlimb	−2	Two stance phases before the first obstacle	NIOHL−2	Left hindlimb
Non-inter-obstacle hindlimb	−1	The stance phase before the first obstacle	NIOHL−1	Left hindlimb
Non-inter-obstacle hindlimb	+1	The stance phase after the second obstacle	NIOHL+1	Left hindlimb

## Appendix A

**Table A1:** Mean values, standard deviations, and significant differences in ground reaction forces between walking over one obstacle and normal walking.

		PFz			IFz			TPFz			Stph		
Limb during Walking over One Obstacle	Limb during Normal Walking	Obstacle: Mean ± SD	Normal: Mean ± SD	*p*-Value	Obstacle: Mean ± SD	Normal: Mean ± SD	*p*-Value	Obstacle: Mean ± SD	Normal: Mean ± SD	*p*-Value	Obstacle: Mean ± SD	Normal: Mean ± SD	*p*-Value
LeFL−1	RFL	62.28 ± 1.67	64.28 ± 1.88	0.222	21.01 ± 3.69	22.84 ± 3.11	0.883	54.42 ± 11.56	50.12 ± 11.09	0.975	0.47 ± 0.08	0.48 ± 0.06	0.999
LeFL+1		64.71 ± 4.03		1.000	25.27 ± 3.79		0.704	55.33 ± 12.32		0.948	0.54 ± 0.08		0.575
LeFL+2		64.45 ± 1.79		1.000	25.94 ± 3.62		0.423	56.11 ± 14.03		0.932	0.55 ± 0.08		0.349
TrFL−1	LFL	64.16 ± 4.21	64.45 ± 2.64	1.000	23.67 ± 3.48	22.67 ± 3.12	0.992	58.13 ± 13.90	49.86 ± 11.71	0.774	0.51 ± 0.08	0.48 ± 0.06	0.980
TrFL+1		65.58 ± 2.17		0.936	24.57 ± 3.41		0.840	57.51 ± 5.99		0.566	0.53 ± 0.08		0.696
LeHL−1	RHL	36.35 ± 4.33	38.01 ± 3.99	0.968	11.97 ± 1.52	12.21 ± 1.06	0.999	42.48 ± 10.23	34.92 ± 6.28	0.473	0.46 ± 0.08	0.45 ± 0.05	1.000
LeHL+1		36.47 ± 4.03		0.973	14.14 ± 1.34		0.041 *	35.42 ± 9.37		1.000	0.53 ± 0.06		0.080
LeHL+2		32.17 ± 3.06		0.269	15.56 ± 0.77		0.012 *	37.74 ± 9.10		0.998	0.63 ± 0.04		0.015 *
TrHL−2	LHL	36.24 ± 4.20	38.14 ± 4.47	0.952	12.34 ± 1.19	12.29 ± 0.85	1.000	37.62 ± 10.51	35.68 ± 7.24	0.999	0.47 ± 0.07	0.45 ± 0.05	0.999
TrHL−1		36.14 ± 4.22		0.939	13.57 ± 1.75		0.438	35.84 ± 5.88		1.000	0.52 ± 0.08		0.240
TrHL+1		35.68 ± 3.82		0.830	13.26 ± 0.95		0.263	41.04 ± 12.74		0.904	0.51 ± 0.06		0.322

Obstacle: mean ± SD = mean and standard deviation during walking over one obstacle; Normal: mean ± SD = mean and standard deviation during normal walking; PFz% = peak vertical force as a percentage of total force; IFz% = vertical impulse as a percentage of total force; TPFz% = time to PFz as a percentage of stance phase duration; SPD% = stance phase duration as a percentage of total stance phase duration; LeFL = leading forelimb; TrFL = trailing forelimb; LeHL = leading hindlimb; TrHL = trailing hindlimb; RFL = right forelimb; LFL = left forelimb; RHL = right hindlimb; LHL = left hindlimb. Significant differences within the groups are marked by an asterisk.

**Table A2:** Mean values, standard deviations, and significant differences in COP values between walking over one obstacle and normal walking.

		COP Med-Lat%			COP Cran-Caud%			COP-Area%			COP-Radius%			COP-Sp		
Limb during Walking over One Obstacle	Limb during Normal Walking	Obstacle: Mean ± SD	Normal: Mean ± SD	*p*-Value	Obstacle: Mean ± SD	Normal: Mean ± SD	*p*-Value	Obstacle: Mean ± SD	Normal: Mean ± SD	*p*-Value	Obstacle: Mean ± SD	Normal: Mean ± SD	*p*-Value	Obstacle: Mean ± SD	Normal: Mean ± SD	*p*-Value
LeFL−1	RFL	6.77 ± 3.53	6.81 ± 2.56	1.000	23.32 ± 3.62	23.01 ± 2.51	1.000	1.04 ± 0.40	1.04 ± 0.31	1.000	0.10 ± 0.02	0.10 ± 0.01	1.000	69.23 ± 9.44	66.31 ± 7.07	0.984
LeFL+1		8.14 ± 3.33		0.947	22.24 ± 3.19		0.996	1.17 ± 0.32		0.967	0.10 ± 0.01		1.000	62.93 ± 8.12		0.949
LeFL+2		7.05 ± 2.79		1.000	22.30 ± 4.45		0.999	1.08 ± 0.34		1.000	0.10 ± 0.02		0.991	57.55 ± 4.75		0.075
TrFL−1	LFL	6.72 ± 2.60	6.78 ± 2.62	1.000	21.03 ± 2.19	23.74 ± 3.95	0.528	0.94 ± 0.31	0.94 ± 0.25	1.000	0.09 ± 0.02	0.10 ± 0.02	0.835	61.57 ± 6.92	66.82 ± 9.63	0.797
TrFL+1		8.13 ± 2.70		0.906	21.31 ± 2.27		0.648	1.27 ± 0.32		0.195	0.09 ± 0.01		0.923	62.27 ± 6.59		0.873
LeHL−1	RHL	6.20 ± 1.39	5.85 ± 1.46	0.998	17.02 ± 3.00	18.70 ± 3.02	0.864	0.76 ± 0.27	0.85 ± 0.28	0.989	0.09 ± 0.01	0.11 ± 0.02	0.501	56.81 ± 4.67	58.99 ± 6.51	0.974
LeHL+1		5.99 ± 1.70		1.000	17.39 ± 2.25		0.921	0.79 ± 0.34		0.999	0.10 ± 0.01		0.777	47.42 ± 4.23		0.007 *
LeHL+2		7.00 ± 2.18		0.978	18.64 ± 2.11		1.000	0.93 ± 0.26		0.999	0.09 ± 0.01		0.369	46.07 ± 3.00		0.012 *
TrHL−2	LHL	6.83 ± 1.75	6.07 ± 1.80	0.958	18.87 ± 2.91	18.30 ± 2.38	0.999	0.90 ± 0.35	0.80 ± 0.24	0.990	0.10 ± 0.02	0.10 ± 0.01	1.000	58.01 ± 6.36	57.08 ± 4.28	1.000
TrHL−1		6.34 ± 1.24		1.000	17.16 ± 2.76		0.950	0.74 ± 0.27		0.998	0.10 ± 0.02		1.000	49.20 ± 4.80		0.023 *
TrHL+1		6.45 ± 1.62		0.999	19.61 ± 2.44		0.878	0.99 ± 0.36		0.799	0.10 ± 0.01		1.000	55.71 ± 4.81		0.993

Obstacle: mean ± SD = mean and standard deviation during walking over one obstacle; Normal: mean ± SD = mean and standard deviation during normal walking; COP = center of pressure; COPmed-lat% = mediolateral displacement of the COP as a percentage of maximum width of paw contact area; COPcran-caud% = craniocaudal displacement of the COP as a percentage of maximum length of paw contact area; COP-Area% = area encompassed by the boundaries of the COP movement as a percentage of paw contact area; COP-Radius% = mean distance of all COP points to the center point of all COP points as a percentage of paw contact area; COP-Sp (mm/s) = speed of COP movement; LeFL = leading forelimb; TrFL = trailing forelimb; LeHL = leading hindlimb; TrHL = trailing hindlimb; RFL = right forelimb; LFL = left forelimb; RHL = right hindlimb; LHL = left hindlimb. Significant differences within groups are marked by an asterisk.

**Table A3:** Mean values, standard deviations, and significant differences in ground reactions forces between walking over two obstacles and normal walking.

		PFz			IFz			TPFz			StPh		
Limb during Walking Over Two Obstacles	Limb during Normal Walking	Obstacle: Mean ± SD	Normal: Mean ± SD	*p*-Value	Obstacle: Mean ± SD	Normal: Mean ± SD	*p*-Value	Obstacle: Mean ± SD	Normal: Mean ± SD	*p*-Value	Obstacle: Mean ± SD	Normal: Mean ± SD	*p*-Value
IOFL−1	RFL	62.60 ± 1.50	64.28 ± 1.88	0.347	20.53 ± 2.44	22.84 ± 3.11	0.538	56.55 ± 12.19	50.12 ± 11.09	0.871	0.45 ± 0.05	0.48 ± 0.06	0.923
IOFL0		64.65 ± 2.70		1.000	25.96 ± 2.30		0.219	67.53 ± 7.49		0.018 *	0.55 ± 0.04		0.164
IOFL+1		65.71 ± 3.10		0.869	26.26 ± 3.05		0.234	58.43 ± 10.26		0.602	0.55 ± 0.06		0.211
NIOFL−1	LFL	64.16 ± 2.94	64.45 ± 2.64	1.000	22.79 ± 2.07	22.67 ± 3.12	1.000	60.77 ± 15.32	49.86 ± 11.71	0.577	0.49 ± 0.04	0.48 ± 0.06	1.000
NIOFL+1		64.08 ± 2.17		1.000	25.77 ± 3.22		0.356	56.15 ± 9.30		0.829	0.55 ± 0.06		0.214
IOHL−1	RHL	36.78 ± 4.67	38.01 ± 3.99	0.994	12.07 ± 1.03	12.21 ± 1.06	1.000	44.13 ± 11.36	34.92 ± 6.28	0.354	0.45 ± 0.05	0.45 ± 0.05	1.000
IOHL0		37.75 ± 5.14		1.000	15.19 ± 1.66		0.007 *	31.98 ± 6.27		0.935	0.55 ± 0.07		0.030 *
IOHL+1		35.98 ± 3.30		0.866	14.09 ± 0.91		0.012	32.91 ± 8.19		0.995	0.53 ± 0.04		0.018 *
NIOHL−2	LHL	36.39 ± 4.05	38.14 ± 4.47	0.964	11.89 ± 1.30	12.29 ± 0.85	0.982	34.34 ± 6.65	35.68 ± 7.24	0.999	0.44 ± 0.06	0.45 ± 0.05	0.999
NIOHL−1		35.79 ± 3.39		0.832	13.52 ± 1.38		0.280	37.96 ± 6.06		0.986	0.52 ± 0.06		0.144
NIOHL+1		36.65 ± 4.81		0.989	13.90 ± 1.00		0.023 *	31.41 ± 5.81		0.767	0.53 ± 0.05		0.047 *

Obstacle: Mean ± SD = mean and standard deviation during walking over two obstacles; Normal: mean ± SD = mean and standard deviation during normal walking; PFz% = peak vertical force as a percentage of total force; IFz% = vertical impulse as a percentage of total force; TPFz% = time to PFz as a percentage of stance phase duration; SPD% = stance phase duration as a percentage of total stance phase duration; IOFL = inter-obstacle forelimb; NIOFL = non-inter-obstacle forelimb; IOHL = inter-obstacle hindlimb; NIOHL = non-inter-obstacle hindlimb; RFL = right forelimb; LFL = left forelimb; RHL = right hindlimb; LHL = left hindlimb. Significant differences within groups are marked by an asterisk.

**Table A4:** Mean values, standard deviations, and significant differences in COP values between walking over two obstacles and normal walking.

		COPmed-lat%			COPcran-caud%			COP-Area%			COP-Radius%			COP-sp		
Limb during Walking over Two Obstacles	Limb during Normal Walking	Obstacle: Mean ± SD	Normal: Mean ± SD	*p*-Value	Obstacle: Mean ± SD	Normal: Mean ± SD	*p*-Value	Obstacle: Mean ± SD	Normal: Mean ± SD	*p*-Value	Obstacle: Mean ± SD	Normal: Mean ± SD	*p*-Value	Obstacle: Mean ± SD	Normal: Mean ± SD	*p*-Value
IOFL−1	RFL	7.00 ± 3.95	6.81 ± 2.56	1.00	22.00 ± 4.83	23.01 ± 2.51	0.997	0.97 ± 0.51	1.04 ± 0.31	1.000	0.10 ± 0.02	0.10 ± 0.01	0.998	67.72 ± 6.86	66.31 ± 7.07	0.999
IOFL0		8.57 ± 4.55		0.932	21.57 ± 3.47		0.932	1.12 ± 0.36		0.998	0.09 ± 0.02		0.777	58.96 ± 4.24		0.155
IOFL+1		7.82 ± 3.21		0.984	22.21 ± 3.00		0.994	1.21 ± 0.41		0.937	0.10 ± 0.02		0.999	59.15 ± 6.96		0.313
NIOFL−1	LFL	7.33 ± 2.73	6.78 ± 2.62	0.999	19.59 ± 2.58	23.74 ± 3.95	0.159	0.95 ± 0.33	0.94 ± 0.25	1.000	0.08 ± 0.02	0.10 ± 0.02	0.376	60.98 ± 6.71	66.82 ± 9.63	0.704
NIOFL+1		8.74 ± 3.08		0.719	21.01 ± 2.48		0.549	1.26 ± 0.34		0.275	0.09 ± 0.01		0.856	60.41 ± 6.25		0.597
IOHL−1	RHL	6.39 ± 1.55	5.85 ± 1.46	0.982	16.62 ± 1.93	18.70 ± 3.02	0.560	0.77 ± 0.34	0.85 ± 0.28	0.995	0.09 ± 0.01	0.11 ± 0.02	0.163	56.38 ± 7.23	58.99 ± 6.51	0.976
IOHL0		5.80 ± 1.59		1.000	16.94 ± 2.97		0.837	0.76 ± 0.26		0.984	0.10 ± 0.02		0.842	45.76 ± 5.96		0.005 *
IOHL+1		6.50 ± 1.83		0.972	20.07 ± 2.45		0.914	0.99 ± 0.33		0.942	0.11 ± 0.02		1.000	53.20 ± 4.01		0.283
NIOHL−2	LHL	6.12 ± 1.98	6.07 ± 1.80	1.000	17.17 ± 2.81	18.30 ± 2.38	0.954	0.86 ± 0.40	0.80 ± 0.24	1.000	0.09 ± 0.02	0.10 ± 0.01	0.692	60.40 ± 9.99	57.08 ± 4.28	0.957
NIOHL−1		6.26 ± 1.64		1.000	16.01 ± 2.32		0.360	0.73 ± 0.14		0.981	0.09 ± 0.01		0.648	51.18 ± 5.13		0.146
NIOHL+1		6.44 ± 1.34		0.998	18.20 ± 3.03		1.000	0.88 ± 0.32		0.996	0.10 ± 0.02		0.980	50.22 ± 5.80		0.107

Obstacle: mean ± SD = mean and standard deviation during walking over two obstacles; Normal: mean ± SD = mean and standard deviation during normal walking; COP = center of pressure; COPmed-lat% = mediolateral displacement of the COP as a percentage of maximum width of paw contact area; COPcran-caud% = craniocaudal displacement of the COP as a percentage of maximum length of paw contact area; COP-Area% = area encompassed by the boundaries of the COP movement as a percentage of paw contact area; COP-Radius% = mean distance of all COP points to the center point of all COP points as a percentage of paw contact area; COP-Sp (mm/s) = speed of COP movement; IOFL = inter-obstacle forelimb; NIOFL = non-inter-obstacle forelimb; IOHL = inter-obstacle hindlimb; NIOHL = non-inter-obstacle hindlimb; RFL = right forelimb; LFL = left forelimb; RHL = right hindlimb; LHL = left hindlimb. Significant differences within groups are marked by an asterisk.

**Table A5:** Mean values, standard deviations, and significant differences in speed and acceleration between walking over one and two obstacles and normal walking.

		Speed			Acceleration		
Limb during Walking over One Obstacle	Limb during Normal Walking	Obstacle: Mean ± SD	Normal: Mean ± SD	*p*-Value	Obstacle: Mean ± SD	Normal: Mean ± SD	*p*-Value
TrFL	LFL	0.94 ± 0.14	1.06 ± 0.12	0.172	0.02 ± 0.03	0.01 ± 0.02	0.723
Limb during walking over two obstacles							
NIOFL	LFL	0.90 ± 0.10	1.06 ± 0.12	0.021 *	0.02 ± 0.02	0.01 ± 0.02	0.687

Obstacle: mean ± SD = mean and standard deviation during walking over one or two obstacles; Normal: mean ± SD = mean and standard deviation during normal walking; TrFL = trailing forelimb; NIOFL = non-inter-obstacle forelimb; LFL = left forelimb. Significant differences within groups are marked by an asterisk.
